# *rac*-[3-Hydroxy-6,9-dimethyl-6-(4-methylpent-3-en-1-yl)-6a,7,8,9,10,10a-hexahydro-6*H*-1,9-epoxybenzo[*c*]chromen-4-yl](phenyl)methanone

**DOI:** 10.1107/S1600536812010756

**Published:** 2012-03-17

**Authors:** Gwendoline Cheng Lian Ee, Soek Sin Teh, Huey Chong Kwong, Mohamed Ibrahim Mohamed Tahir, Siau Hui Mah

**Affiliations:** a Department Chemistry, Faculty Science, University Putra Malaysia, 43400 UPM Serdang, Selangor, Malaysia; b Department Chemistry, Faculty Science, Universiti Putra Malaysia, 43400 UPM Serdang, Selangor, Malaysia

## Abstract

The title compound congestiflorone, C_28_H_32_O_4_, which was isolated from the stem bark of *Mesua congestiflora*, consists of a benzophenone skeleton with two attached pyran rings to which a cyclo­hexane ring and a C6 side chain are bonded. The benzene ring is significantly distorted from planarity (r.m.s. deviation = 0.0007 Å) due to the constraints imposed by junctions with the two pyran rings. The cyclo­hexane ring is in a chair conformation, one pyran ring is in a boat conformation, while the other is a distorted chair. The phenyl and benzene rings make a dihedral angle of 55.85 (9)°. An intra­molecular O—H⋯O hydrogen bond is observed. In the crystal, mol­ecules are linked *via* C—H⋯O inter­actions.

## Related literature

For phytochemical investigations of *Mesua congestiflora*, see: Awang *et al.* (2010 ▶); Bala & Seshadri (1971 ▶); Ee *et al.* (2005*b* ▶); Bandaranayak *et al.* (1975 ▶); Morel *et al.* (1999 ▶); Walia & Mukerjee (1984 ▶). For the biological activity of *Congestiflora* species, see: Pinto *et al.* (1994 ▶); Ee *et al.* (2005*a* ▶); Mazumder *et al.* (2004 ▶); Verotta *et al.* (2004 ▶); Huerta-Reyes *et al.* (2004 ▶). For related structures, see: Hua *et al.* (2008 ▶); Liu *et al.* (2005 ▶). For a description of the Cambridge Structural Database, see Allen (2002 ▶)
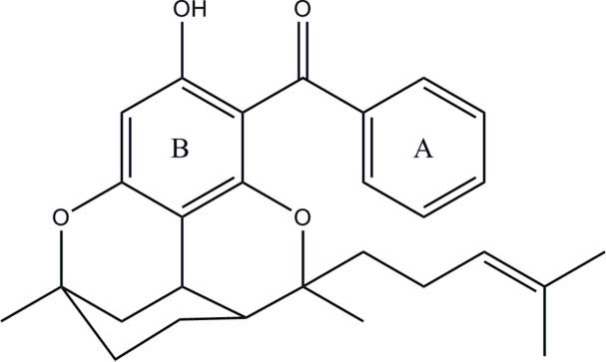


## Experimental

### 

#### Crystal data

C_28_H_32_O_4_
*M* *_r_* = 432.56
Triclinic, 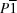

*a* = 6.2022 (4) Å
*b* = 7.5220 (4) Å
*c* = 24.7673 (15) Å
α = 98.410 (5)°
β = 94.425 (5)°
γ = 94.200 (5)°
*V* = 1135.43 (12) Å^3^
*Z* = 2
Cu *K*α radiation
μ = 0.66 mm^−1^
*T* = 150 K
0.29 × 0.09 × 0.05 mm

#### Data collection

Oxford Diffraction Gemin area-detector diffractometer
Absorption correction: multi-scan (*CrysAlis PRO*; Oxford Diffraction, 2006 ▶) *T*_min_ = 0.942, *T*_max_ = 0.968
15011 measured reflections
4340 independent reflections
3423 reflections with *I* > 2σ(*I*)
*R*_int_ = 0.034

#### Refinement

*R*[*F* ^2^ > 2σ(*F* ^2^)] = 0.046
*wR*(*F* ^2^) = 0.117
*S* = 1.00
4321 reflections
289 parameters
H-atom parameters constrained
Δρ_max_ = 0.39 e Å^−3^
Δρ_min_ = −0.34 e Å^−3^

### 

Data collection: *CrysAlis CCD* (Oxford Diffraction, 2006 ▶); cell refinement: *CrysAlis CCD*; data reduction: *CrysAlis RED* (Oxford Diffraction, 2006 ▶); program(s) used to solve structure: *SIR92* (Altomare *et al.*, 1994 ▶); program(s) used to refine structure: *CRYSTALS* (Betteridge *et al.*, 2003 ▶); molecular graphics: *Mercury* (Macrae *et al.*, 2006 ▶); software used to prepare material for publication: *CRYSTALS*.

## Supplementary Material

Crystal structure: contains datablock(s) global, I. DOI: 10.1107/S1600536812010756/kp2392sup1.cif

Structure factors: contains datablock(s) I. DOI: 10.1107/S1600536812010756/kp2392Isup2.hkl

Additional supplementary materials:  crystallographic information; 3D view; checkCIF report

## Figures and Tables

**Table 1:** Hydrogen-bond geometry (Å, °)

*D*—H⋯*A*	*D*—H	H⋯*A*	*D*⋯*A*	*D*—H⋯*A*
C18—H181⋯O1^i^	1.00	2.60	3.563 (3)	162
O11—H111⋯O1	0.87	1.78	2.551 (3)	145

Symmetry code: (i) 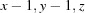.

**Fig. 1.**
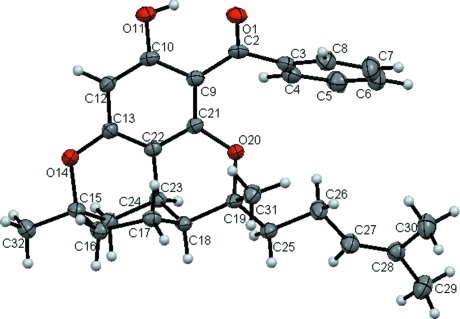
The molecular structure of the title compound shows a 50% probability displacement ellipsoids and the atom-numbering scheme. The intramolecular hydrogen bond O11-H···O1 is observed.

**Fig. 2.**
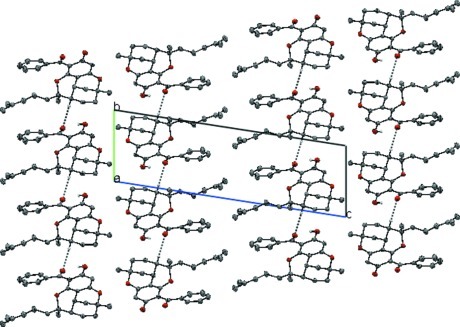
The crystal packing of the title compound is viewed along the a axis. H atoms not involved in hydrogen bonds have been omitted for clarity. Hydrohen bonds are shown in dashed lines.

## supplementary crystallographic information

### Comment

*Mesua congestiflora* is native to Indonesia and is also distributed throughout Borneo, Sarawak. Previous phytochemical investigations on the genus show the existence of xanthones (Bandaranayak *et al.* 1975; Walia & Mukerjee 1984; Ee *et al.* 2005*b*), coumarins (Bandaranayak, Selliah *et al.* 1975; Morel, Guilet *et al.* 1999; Awang, Chan *et al.* 2010), terpenoids (Ee *et al.* 2005*a*) and essential oils (Bala & Seshadri 1971). These secondary metabolites have been extensively reported for their biological activities; for instance antifungal(Pinto *et al.* 1994), anticancer(Ee *et al.* 2005*a*), antibacterial (Mazumder, Dastidar *et al.* 2004; Verotta, Lovaglio *et al.* 2004), and anti-HIV-1(Huerta-Reyes *et al.* 2004). However, pharmacognosy and preliminary phytochemical analysis on this species have not been reported before.

The title compound (I), congestiflorone C_28_H_32_O_4_ skeleton comprises five 6-membered rings and a 1-methylpent-2-enyl side chain (Fig. 1). The skeleton is similar to that of Sumadain A (Hua, Wang *et al.* 2008) except for the absence of 2 methylene groups next to the carbonyl group. Dihedral angle of those two benzene rings was 55.85 (9)°. The benzene ring (C9—C10—C12—C13—C21—C22) is not planar (the largest deviation from the best least squares plane is 0.082 (2) Å at C22). This departure from planarity of the ring A might be caused by the constraint of two adjacent pyrane rings which adapt a distorted chair conformation and a boat conformation. The cyclohexane ring adapted a chair conformation and the puckering parameter is Q= 0.5635 (18), θ= 166.13 (18)°, Φ2= 93.4 (8)°. The conformations of pyran and cyclohexane rings are comparable to the structure of Sumadain A (Hua, Wang *et al.* 2008). The orientation of the 1-methylpent-2-enly (C25—C28) side chain with respect to the cyclohexane ring is indicated by the torsion angle of C19—C25—C26—C27 = 177.17 (16)° and C25—C26—C27—C28=144.0 (2)° [169.5 (3)° and 145.4 (4)° respectively in Hua, Wang *et al.* 2008]. The structure of the molecule exhibits an intramolecular O—H···O hydrogen bond (Table 1). In the crystal, molecules are linked *via* a intermolecular C—H···O hydrogen bonding in the a,b-plane (Fig. 2).

The cystallographic data of this crystal structure has been deposited at Cambridge Crystallographic Data Center with deposition number CCDC 849099. (Allen, 2002)

### Experimental

The stem bark of *Mesua congestiflora* was collected from the Sri Aman district in Sarawak, Malaysia. The sample (840 g) was milled, air-dried and ground, the powdered sample was extracted with *n*-hexane. The extract was dried under reduced pressure in a rotary evaporator to yield the hexane extract (5.50 g). Stepwise gradient systems using hexane/chloroform and chloroform/methanol or hexane/ethyl acetate and ethyl acetate/methanol, were applied for the separation and purification of the extract. Congestiflorone, a yellowish crystals with the melting point of 483 K were isolated. This compound was crystallised from slow evaporation of ethyl acetate at room temperature.

### Refinement

The H atoms were all located in a difference map, but those attached to carbon atoms were repositioned geometrically. The H atoms were initially refined with soft restraints on the bond lengths and angles to regularize their geometry (C—H in the range 0.93–0.98, N—H in the range 0.86–0.89 N—H to 0.86 O—H = 0.82 Å) and *U*_iso_(H) (in the range 1.2–1.5 times *U*_eq_ of the parent atom), after which the positions were refined with riding constraints.

### Crystal data

**Table:**

C_28_H_32_O_4_	*Z* = 2
*M**_r_* = 432.56	*F*(000) = 464
Triclinic, *P*1	*D*_x_ = 1.265 Mg m^−^^3^
Hall symbol: -P 1	Melting point: 483 K
*a* = 6.2022 (4) Å	Cu *K*α radiation, λ = 1.54180 Å
*b* = 7.5220 (4) Å	Cell parameters from 4627 reflections
*c* = 24.7673 (15) Å	θ = 4–71°
α = 98.410 (5)°	µ = 0.66 mm^−^^1^
β = 94.425 (5)°	*T* = 150 K
γ = 94.200 (5)°	Needle, yellow
*V* = 1135.43 (12) Å^3^	0.29 × 0.09 × 0.05 mm

### Data collection

**Table:**

Oxford Diffraction Gemin area-detector diffractometer	4340 independent reflections
Radiation source: sealed x-ray tube	3423 reflections with *I* > 2σ(*I*)
Graphite monochromator	*R*_int_ = 0.034
ω/2θ scans	θ_max_ = 71.2°, θ_min_ = 3.6°
Absorption correction: multi-scan (*CrysAlis PRO*; Oxford Diffraction, 2006)	*h* = −7→7
*T*_min_ = 0.942, *T*_max_ = 0.968	*k* = −9→9
15011 measured reflections	*l* = −30→30

### Refinement

**Table:**

Refinement on *F*^2^	Primary atom site location: structure-invariant direct methods
Least-squares matrix: full	Hydrogen site location: inferred from neighbouring sites
*R*[*F*^2^ > 2σ(*F*^2^)] = 0.046	H-atom parameters constrained
*wR*(*F*^2^) = 0.117	Method = Modified Sheldrick *w* = 1/[σ^2^(*F*^2^) + ( 0.05*P*)^2^ + 0.61*P*], where *P* = (max(*F*_o_^2^,0) + 2*F*_c_^2^)/3
*S* = 1.00	(Δ/σ)_max_ = 0.001
4321 reflections	Δρ_max_ = 0.39 e Å^−^^3^
289 parameters	Δρ_min_ = −0.34 e Å^−^^3^
0 restraints	

### Special details

**Table:**

Refinement. For this compound, 15011 numbers of reflections were collected and measured during the refinement. Symmetry related reflections were measured more than once and after merging the symmetry equivalent reflections there were only 4340 reflection left. 19 more reflections were filtered, as σ cutoff was set as -3 and (sin*θ*/*x*)set to>0.01 (to eliminate reflection measured near the vicinity of beam stop) therefore numbers of reflection reduced to 4321.

### Fractional atomic coordinates and isotropic or equivalent isotropic displacement parameters (Å^2^)

**Table:**

	*x*	*y*	*z*	*U*_iso_*/*U*_eq_	
O1	1.3916 (2)	0.62936 (17)	0.77877 (5)	0.0320	
C2	1.2067 (3)	0.5510 (2)	0.76653 (7)	0.0250	
C3	1.1384 (3)	0.4954 (2)	0.70701 (7)	0.0263	
C4	0.9367 (3)	0.5313 (2)	0.68404 (8)	0.0291	
C5	0.8872 (3)	0.4939 (3)	0.62773 (8)	0.0365	
C6	1.0351 (4)	0.4162 (3)	0.59461 (8)	0.0431	
C7	1.2346 (4)	0.3778 (3)	0.61725 (9)	0.0447	
C8	1.2880 (3)	0.4204 (3)	0.67332 (8)	0.0355	
C9	1.0615 (3)	0.5227 (2)	0.80880 (7)	0.0235	
C10	1.1140 (3)	0.6170 (2)	0.86326 (7)	0.0255	
O11	1.2907 (2)	0.73655 (17)	0.87537 (5)	0.0336	
C12	0.9944 (3)	0.5844 (2)	0.90629 (7)	0.0267	
C13	0.8146 (3)	0.4582 (2)	0.89609 (7)	0.0239	
O14	0.7243 (2)	0.40329 (16)	0.93968 (5)	0.0282	
C15	0.5848 (3)	0.2316 (2)	0.92940 (7)	0.0255	
C16	0.7221 (3)	0.0698 (2)	0.91977 (7)	0.0263	
C17	0.8289 (3)	0.0419 (2)	0.86545 (7)	0.0251	
C18	0.6761 (3)	0.0648 (2)	0.81520 (7)	0.0224	
C19	0.7858 (3)	0.0839 (2)	0.76156 (7)	0.0235	
O20	0.82634 (19)	0.27776 (16)	0.75412 (5)	0.0252	
C21	0.8751 (3)	0.3983 (2)	0.80136 (7)	0.0220	
C22	0.7405 (3)	0.3818 (2)	0.84248 (7)	0.0222	
C23	0.5588 (3)	0.2361 (2)	0.82988 (7)	0.0217	
C24	0.4288 (3)	0.2301 (2)	0.87914 (7)	0.0252	
C25	0.6276 (3)	0.0004 (2)	0.71244 (7)	0.0263	
C26	0.6933 (3)	0.0294 (3)	0.65592 (8)	0.0363	
C27	0.5303 (3)	−0.0667 (3)	0.61092 (8)	0.0320	
C28	0.5695 (3)	−0.1476 (3)	0.56176 (7)	0.0294	
C29	0.3899 (3)	−0.2423 (3)	0.52118 (8)	0.0404	
C30	0.7924 (3)	−0.1539 (3)	0.54199 (9)	0.0430	
C31	1.0059 (3)	0.0067 (3)	0.75961 (8)	0.0304	
C32	0.4671 (3)	0.2328 (3)	0.98082 (8)	0.0319	
H41	0.8314	0.5832	0.7073	0.0367*	
H51	0.7488	0.5234	0.6122	0.0454*	
H61	0.9999	0.3875	0.5557	0.0533*	
H71	1.3367	0.3232	0.5942	0.0547*	
H81	1.4295	0.3981	0.6891	0.0457*	
H121	1.0361	0.6440	0.9423	0.0345*	
H161	0.8374	0.0796	0.9503	0.0335*	
H162	0.6217	−0.0382	0.9209	0.0328*	
H171	0.9603	0.1294	0.8679	0.0317*	
H172	0.8805	−0.0812	0.8599	0.0324*	
H181	0.5662	−0.0425	0.8070	0.0291*	
H231	0.4619	0.2584	0.7977	0.0269*	
H241	0.3411	0.3350	0.8846	0.0319*	
H242	0.3299	0.1190	0.8740	0.0317*	
H251	0.6091	−0.1324	0.7137	0.0342*	
H252	0.4852	0.0508	0.7176	0.0341*	
H261	0.8365	−0.0145	0.6505	0.0461*	
H262	0.7055	0.1603	0.6535	0.0467*	
H271	0.3814	−0.0696	0.6200	0.0408*	
H291	0.3915	−0.1929	0.4867	0.0632*	
H292	0.4065	−0.3713	0.5141	0.0633*	
H293	0.2496	−0.2260	0.5358	0.0628*	
H301	0.7949	−0.1071	0.5070	0.0677*	
H302	0.8301	−0.2777	0.5363	0.0685*	
H303	0.9008	−0.0786	0.5679	0.0685*	
H311	1.0584	0.0141	0.7238	0.0482*	
H312	1.1094	0.0772	0.7879	0.0485*	
H313	0.9902	−0.1190	0.7658	0.0489*	
H323	0.3689	0.1245	0.9770	0.0491*	
H322	0.5668	0.2357	1.0134	0.0499*	
H321	0.3815	0.3378	0.9856	0.0494*	
H111	1.3748	0.7225	0.8486	0.0530*	

### Atomic displacement parameters (Å^2^)

**Table:**

	*U*^11^	*U*^22^	*U*^33^	*U*^12^	*U*^13^	*U*^23^
O1	0.0270 (7)	0.0331 (7)	0.0343 (7)	−0.0087 (6)	0.0017 (5)	0.0060 (6)
C2	0.0252 (9)	0.0171 (9)	0.0327 (10)	−0.0010 (7)	0.0009 (7)	0.0060 (7)
C3	0.0282 (9)	0.0220 (9)	0.0289 (9)	−0.0045 (7)	0.0043 (7)	0.0067 (7)
C4	0.0316 (10)	0.0255 (10)	0.0307 (10)	−0.0008 (8)	0.0052 (8)	0.0064 (8)
C5	0.0369 (11)	0.0391 (12)	0.0333 (11)	−0.0020 (9)	−0.0017 (8)	0.0103 (9)
C6	0.0479 (13)	0.0531 (14)	0.0259 (10)	−0.0068 (11)	0.0022 (9)	0.0043 (9)
C7	0.0419 (12)	0.0568 (14)	0.0341 (11)	0.0006 (10)	0.0143 (9)	−0.0010 (10)
C8	0.0297 (10)	0.0410 (12)	0.0356 (11)	0.0000 (9)	0.0073 (8)	0.0046 (9)
C9	0.0252 (9)	0.0196 (9)	0.0257 (9)	0.0003 (7)	0.0007 (7)	0.0045 (7)
C10	0.0263 (9)	0.0182 (9)	0.0308 (10)	−0.0024 (7)	−0.0020 (7)	0.0047 (7)
O11	0.0342 (7)	0.0309 (7)	0.0319 (7)	−0.0133 (6)	−0.0005 (6)	0.0020 (6)
C12	0.0331 (10)	0.0213 (9)	0.0233 (9)	−0.0015 (8)	−0.0005 (7)	−0.0012 (7)
C13	0.0263 (9)	0.0201 (9)	0.0254 (9)	0.0025 (7)	0.0052 (7)	0.0020 (7)
O14	0.0326 (7)	0.0272 (7)	0.0227 (6)	−0.0052 (5)	0.0049 (5)	−0.0005 (5)
C15	0.0246 (9)	0.0251 (9)	0.0256 (9)	−0.0033 (7)	0.0036 (7)	0.0024 (7)
C16	0.0254 (9)	0.0280 (10)	0.0257 (9)	−0.0002 (7)	−0.0001 (7)	0.0071 (7)
C17	0.0241 (9)	0.0220 (9)	0.0295 (9)	0.0022 (7)	0.0025 (7)	0.0050 (7)
C18	0.0211 (8)	0.0197 (9)	0.0254 (9)	−0.0023 (7)	0.0034 (7)	0.0014 (7)
C19	0.0224 (9)	0.0199 (9)	0.0264 (9)	−0.0036 (7)	0.0031 (7)	0.0003 (7)
O20	0.0300 (7)	0.0226 (6)	0.0213 (6)	−0.0055 (5)	0.0033 (5)	0.0009 (5)
C21	0.0234 (8)	0.0189 (9)	0.0228 (9)	0.0008 (7)	−0.0009 (7)	0.0025 (7)
C22	0.0224 (8)	0.0188 (9)	0.0254 (9)	0.0026 (7)	0.0010 (7)	0.0035 (7)
C23	0.0191 (8)	0.0231 (9)	0.0228 (9)	0.0004 (7)	0.0006 (7)	0.0039 (7)
C24	0.0220 (9)	0.0269 (9)	0.0266 (9)	0.0012 (7)	0.0033 (7)	0.0037 (7)
C25	0.0247 (9)	0.0247 (9)	0.0270 (9)	−0.0038 (7)	0.0030 (7)	−0.0013 (7)
C26	0.0315 (10)	0.0470 (13)	0.0264 (10)	−0.0084 (9)	0.0046 (8)	−0.0027 (9)
C27	0.0267 (9)	0.0387 (11)	0.0291 (10)	−0.0007 (8)	0.0037 (8)	0.0011 (8)
C28	0.0323 (10)	0.0303 (10)	0.0254 (9)	0.0032 (8)	0.0012 (8)	0.0040 (8)
C29	0.0416 (12)	0.0440 (13)	0.0321 (11)	0.0039 (10)	−0.0013 (9)	−0.0041 (9)
C30	0.0398 (12)	0.0566 (14)	0.0316 (11)	0.0061 (10)	0.0092 (9)	−0.0002 (10)
C31	0.0249 (9)	0.0349 (11)	0.0296 (10)	0.0009 (8)	0.0043 (7)	−0.0015 (8)
C32	0.0332 (10)	0.0350 (11)	0.0277 (10)	−0.0017 (8)	0.0076 (8)	0.0050 (8)

### Geometric parameters (Å, º)

**Table:**

O1—C2	1.245 (2)	C18—C23	1.537 (2)
C2—C3	1.492 (3)	C18—H181	1.004
C2—C9	1.461 (2)	C19—O20	1.502 (2)
C3—C4	1.395 (3)	C19—C25	1.530 (2)
C3—C8	1.391 (3)	C19—C31	1.523 (2)
C4—C5	1.388 (3)	O20—C21	1.370 (2)
C4—H41	0.969	C21—C22	1.379 (2)
C5—C6	1.382 (3)	C22—C23	1.494 (2)
C5—H51	0.967	C23—C24	1.518 (2)
C6—C7	1.386 (3)	C23—H231	1.003
C6—H61	0.961	C24—H241	0.990
C7—C8	1.387 (3)	C24—H242	0.986
C7—H71	0.959	C25—C26	1.529 (2)
C8—H81	0.969	C25—H251	1.002
C9—C10	1.431 (2)	C25—H252	0.996
C9—C21	1.416 (2)	C26—C27	1.506 (3)
C10—O11	1.351 (2)	C26—H261	0.982
C10—C12	1.385 (2)	C26—H262	0.993
O11—H111	0.874	C27—C28	1.328 (3)
C12—C13	1.393 (2)	C27—H271	0.967
C12—H121	0.945	C28—C29	1.503 (3)
C13—O14	1.357 (2)	C28—C30	1.504 (3)
C13—C22	1.396 (2)	C29—H291	0.981
O14—C15	1.479 (2)	C29—H292	0.975
C15—C16	1.536 (2)	C29—H293	0.977
C15—C24	1.515 (2)	C30—H301	0.982
C15—C32	1.515 (2)	C30—H302	0.970
C16—C17	1.539 (2)	C30—H303	0.978
C16—H161	0.991	C31—H311	0.976
C16—H162	0.992	C31—H312	0.978
C17—C18	1.543 (2)	C31—H313	0.979
C17—H171	1.001	C32—H323	0.969
C17—H172	0.996	C32—H322	0.975
C18—C19	1.559 (2)	C32—H321	0.982
			
O1—C2—C3	116.89 (16)	O20—C19—C31	105.61 (13)
O1—C2—C9	120.99 (16)	C25—C19—C31	112.13 (14)
C3—C2—C9	122.07 (15)	C19—O20—C21	115.62 (12)
C2—C3—C4	121.53 (16)	C9—C21—O20	121.81 (15)
C2—C3—C8	118.50 (16)	C9—C21—C22	122.22 (16)
C4—C3—C8	119.73 (17)	O20—C21—C22	115.80 (15)
C3—C4—C5	119.91 (18)	C13—C22—C21	118.67 (16)
C3—C4—H41	120.2	C13—C22—C23	122.24 (15)
C5—C4—H41	119.9	C21—C22—C23	116.14 (15)
C4—C5—C6	120.00 (19)	C18—C23—C22	103.24 (13)
C4—C5—H51	119.1	C18—C23—C24	112.94 (14)
C6—C5—H51	120.9	C22—C23—C24	110.36 (14)
C5—C6—C7	120.37 (19)	C18—C23—H231	110.6
C5—C6—H61	120.2	C22—C23—H231	109.6
C7—C6—H61	119.4	C24—C23—H231	109.9
C6—C7—C8	119.9 (2)	C23—C24—C15	108.64 (14)
C6—C7—H71	120.2	C23—C24—H241	110.7
C8—C7—H71	119.9	C15—C24—H241	109.7
C3—C8—C7	120.02 (19)	C23—C24—H242	110.5
C3—C8—H81	119.9	C15—C24—H242	108.7
C7—C8—H81	120.1	H241—C24—H242	108.4
C2—C9—C10	119.22 (15)	C19—C25—C26	116.47 (15)
C2—C9—C21	124.97 (16)	C19—C25—H251	106.8
C10—C9—C21	115.68 (15)	C26—C25—H251	108.8
C9—C10—O11	120.83 (16)	C19—C25—H252	107.8
C9—C10—C12	122.15 (16)	C26—C25—H252	108.3
O11—C10—C12	116.94 (16)	H251—C25—H252	108.4
C10—O11—H111	109.2	C25—C26—C27	111.43 (16)
C10—C12—C13	119.00 (16)	C25—C26—H261	109.8
C10—C12—H121	120.7	C27—C26—H261	109.3
C13—C12—H121	120.2	C25—C26—H262	109.7
C12—C13—O14	118.03 (15)	C27—C26—H262	109.1
C12—C13—C22	120.50 (16)	H261—C26—H262	107.4
O14—C13—C22	121.31 (16)	C26—C27—C28	127.51 (18)
C13—O14—C15	117.10 (13)	C26—C27—H271	114.7
O14—C15—C16	110.88 (14)	C28—C27—H271	117.8
O14—C15—C24	109.24 (14)	C27—C28—C29	121.68 (18)
C16—C15—C24	108.97 (14)	C27—C28—C30	123.77 (18)
O14—C15—C32	103.92 (14)	C29—C28—C30	114.55 (17)
C16—C15—C32	112.01 (15)	C28—C29—H291	110.1
C24—C15—C32	111.74 (15)	C28—C29—H292	110.3
C15—C16—C17	116.82 (14)	H291—C29—H292	109.4
C15—C16—H161	109.1	C28—C29—H293	109.9
C17—C16—H161	108.3	H291—C29—H293	109.0
C15—C16—H162	105.6	H292—C29—H293	108.0
C17—C16—H162	108.1	C28—C30—H301	110.0
H161—C16—H162	108.7	C28—C30—H302	109.5
C16—C17—C18	113.52 (14)	H301—C30—H302	108.5
C16—C17—H171	109.4	C28—C30—H303	111.4
C18—C17—H171	108.8	H301—C30—H303	107.1
C16—C17—H172	108.9	H302—C30—H303	110.3
C18—C17—H172	109.1	C19—C31—H311	109.0
H171—C17—H172	106.9	C19—C31—H312	109.6
C17—C18—C19	116.21 (14)	H311—C31—H312	108.8
C17—C18—C23	108.18 (14)	C19—C31—H313	109.2
C19—C18—C23	106.99 (13)	H311—C31—H313	110.3
C17—C18—H181	108.4	H312—C31—H313	109.9
C19—C18—H181	107.7	C15—C32—H323	109.1
C23—C18—H181	109.2	C15—C32—H322	112.2
C18—C19—O20	111.90 (13)	H323—C32—H322	108.1
C18—C19—C25	108.74 (14)	C15—C32—H321	109.6
O20—C19—C25	104.37 (13)	H323—C32—H321	108.5
C18—C19—C31	113.70 (14)	H322—C32—H321	109.3

### Hydrogen-bond geometry (Å, º)

**Table:**

*D*—H···*A*	*D*—H	H···*A*	*D*···*A*	*D*—H···*A*
C18—H181···O1^i^	1.00	2.60	3.563 (3)	162
O11—H111···O1	0.87	1.78	2.551 (3)	145

Symmetry code: (i) *x*−1, *y*−1, *z*.

## References

[bb1] Allen, F. H. (2002). *Acta Cryst.* B**58**, 380–388. 10.1107/s0108768102003890 12037359

[bb2] Altomare, A., Cascarano, G., Giacovazzo, C., Guagliardi, A., Burla, M. C., Polidori, G. & Camalli, M. (1994). *J. Appl. Cryst.* **27**, 435.

[bb3] Awang, K., Chan, G., Litaudon, M., Ismail, N. H., Martin, M. T. & Gueritte, F. (2010). *Bioorg. Med. Chem.* **18**, 7873–7877. 10.1016/j.bmc.2010.09.044 20943395

[bb4] Bala, K. R. & Seshadri, T. R. (1971). *Phytochemistry*, **10**, 1131–1134.

[bb5] Bandaranayak, W. M., Selliah, S. S. & Sultanbawa, M. U. S. (1975). *Phytochemistry*, **14**, 265–269.

[bb6] Betteridge, P. W., Carruthers, J. R., Cooper, R. I., Prout, K. & Watkin, D. J. (2003). *J. Appl. Cryst.* **36**, 1487.

[bb7] Ee, G. C. L., Lim, C. K., Cheow, Y. L. & Sukari, M. A. (2005*a*). *Malays. J. Sci.* **24**, 183–185.

[bb8] Ee, G. C. L., Lim, C. K., Rahmat, A. & Lee, H. L. (2005*b*). *Trop. Biomed.* **22.**, 99–102. 16883274

[bb9] Hua, S.-Z., Wang, X.-B., Luo, J.-G., Wang, J.-S. & Kong, L.-Y. (2008). *Tetrahedron Lett.* **49**, 5658–5661.

[bb10] Huerta-Reyes, M., Basualdo Mdel, C., Abe, F., Jimenez-Estrada, M., Soler, C. & Reyes-Chilpa, R. (2004). *Biol. Pharm. Bull.* **27.**, 1471–1475. 10.1248/bpb.27.1471 15340243

[bb11] Liu, K.-Y., Gao, W.-Y., Zhang, T.-J., Chen, H.-X. & Zhou, B. (2005). *Acta Cryst.* E**61**, o391–o392.

[bb12] Macrae, C. F., Edgington, P. R., McCabe, P., Pidcock, E., Shields, G. P., Taylor, R., Towler, M. & van de Streek, J. (2006). *J. Appl. Cryst.* **39**, 453–457.

[bb13] Mazumder, R., Dastidar, S. G., Basu, S. P., Mazumder, A. & Singh, S. K. (2004). *Phytother. Res.* **18**, 824–826. 10.1002/ptr.1572 15551387

[bb14] Morel, C., Guilet, D., Oger, J. M., Seraphin, D., Sevenet, T., Wiart, C., Hadi, A. H. A., Richomme, P. & Bruneton, J. (1999). *Phytochemistry*, **50.**, 1243–1247.

[bb15] Oxford Diffraction (2006). *CrysAlis CCD* and *CrysAlis RED* Oxford Diffraction Ltd, Abingdon, England.

[bb16] Pinto, D. C. G., Fuzzati, N., Pazmino, X. C. & Hostettmann, K. (1994). *Phytochemistry*, **3** *.* 875-878. 10.1016/s0031-9422(00)90375-3 7765695

[bb17] Verotta, L., Lovaglio, E., Vidari, G., Finzi, P. V., Neri, M. G., Raimondi, A., Parapini, S., Taramelli, D., Riva, A. & Bombardelli, E. (2004). *Phytochemistry*, **65**, 2867–2879. 10.1016/j.phytochem.2004.07.001 15501254

[bb18] Walia, S. & Mukerjee, S. K. (1984). *Phytochemistry*, **23**, 1816–1817.

